# Ketogenic drug tricaprilin (CER-0001) for the treatment of refractory infantile epileptic spasms: a phase 1/2a study

**DOI:** 10.3389/fped.2025.1575014

**Published:** 2026-02-20

**Authors:** Marc Cantillon, Sylvia Chen, Nikki McIntyre, Samuel T. Henderson, Kate Riney, Derrick W. S. Chan, John Lawson, Lilian Chow

**Affiliations:** 1 Robert Wood Johnson Medical School, New Brunswick, NJ, United States; 2 Cerecin Australia Pty Ltd, Melbourne, VIC, Australia; 3 Neurosciences Unit, Queensland Children's Hospital, South Brisbane, QLD, Australia; 4 School of Medicine, University of Queensland, St Lucia, QLD, Australia; 5 Paediatric Neurology, KK Women's and Children's Hospital Duke-NUS Medical School, Singapore, Singapore; 6 Department of Neurology, Sydney Children's Hospital, High Street Randwick, Sydney, NSW, Australia

**Keywords:** epilepsy, infantile spasms, ketogenic, seizures, tricaprilin

## Abstract

**Objective:**

Ketogenic diets (KDs) have been used in the management of multiple pediatric and adult epilepsies. CER-0001 (tricaprilin) is an orally administered, liquid, investigational ketogenic drug with preclinical efficacy in an infantile spasms (IS) animal model. This study aims to evaluate the safety and tolerability of CER-0001 for the treatment of drug-resistant IS in individuals not following a KD.

**Methods:**

Participants underwent a 28-day study period following baseline measurements that included a 24 h video electroencephalogram (vEEG) and caregiver diary. CER-0001 was titrated over a period of up to 14 days, with the total daily dose divided into four doses every 6 h to determine the maximum tolerated dose or to achieve seizure control. Participants who experienced improved seizure control were maintained on the dose for 7 days, while those without clinical benefit underwent a repeat vEEG on the final day of titration to confirm continuation to the maintenance phase. A 1-year open-label extension phase was offered to participants in Australia who exhibited improvement in seizure control.

**Results:**

Out of the eight participants enrolled in the study, it was found that the majority of reported treatment emergent adverse events (TEAEs) were mild and moderate. The most common TEAEs were gastrointestinal (GI) related, observed in 75% of participants, with vomiting reported in 50%. Two participants (25%) experienced severe TEAEs. Four participants showed a 50%–75% improvement in seizure clusters on the vEEG, while two experienced a 100% improvement. Of the eight participants who completed the titration phase, seven continued into the maintenance phase of the study, and of these, three continued CER-0001 treatment beyond completion of the maintenance phase, either through the open-label extension protocol in Australia (n = 2), or through the special access route (SAR) program in Singapore (n = 1). Overall, CER-0001 was well tolerated and was associated with preliminary benefits for refractory IS.

**Significance:**

Our findings demonstrate that CER-0001 offers a novel potential treatment option as a prescription drug for ketogenic therapy, without the restrictions imposed by KDs.

## Highlights

The study showed that CER-0001 had a good safety profile when administered daily for the treatment of infantile epileptic spasms.
The results demonstrated efficacy in a clinical setting, indicating promising outcomes for a ketogenic mechanism.
Overall, CER-0001 was generally well tolerated; the most common side effects were GI in some participants.
CER-0001 may offer a novel treatment for pediatric epilepsy without the need for a ketogenic diet.

## Introduction

Infantile epileptic spasms (IS) are a specific seizure type observed during infancy, typically occurring between 1 and 24 months of age. They are the characteristic seizure type seen in infantile epileptic spasms syndrome (IESS), a clinical syndrome with developmental and epileptic encephalopathy (DEE), epileptic spasms, hypsarrhythmia or focal/multifocal epileptiform abnormality, and developmental stagnation or regression (1). West syndrome is a subgroup of IESS where hypsarrhythmia is observed on EEG.

Infantile epileptic spasms occur more frequently upon waking, happening in clusters of variable duration, with each seizure lasting only a few seconds. Infantile epileptic spasms are typically diagnosed between 3 and 8 months of age, with most children showing developmental disabilities later in life and potentially developing other types of seizures or epilepsy. The prevailing viewpoint among experts is that the goal of treatment should be controlling the IS, both clinically and electrophysiologically (2). First-line treatments for IS are hormonal therapy with adrenocorticotropic hormone (ACTH) or prednisolone, or the use of the gamma aminobutyric acid (GABA)-transaminase inhibitor, vigabatrin (3). Unfortunately, 40%–60% of patients fail to respond to these first-line treatments, which may lead to the development of other seizure types (4–6). The ketogenic diet (KD) has been used since the 1920s to treat many kinds of epilepsies (7). In a systematic review of KD studies, it was found that 64.7% of the participants experienced a median rate of seizure reduction of more than 50%, with a median of 34.6% achieving freedom from epileptic spasms (8). Moreover, a randomized controlled trial conducted in refractory epilepsy in patients aged 1–18 years showed that patients on KDs experienced a three-time higher decrease in seizure severity score after 6 weeks of treatment compared with the standard of care (9).

CER-0001 is an eight-carbon, medium chain triglyceride (MCT) that can induce ketosis without the need for dietary restriction. CER-0001 is an investigational drug being studied in Alzheimer's disease, migraine, and seizure disorders. Because of their physiochemical characteristics, MCTs can induce ketosis upon oral administration without the need for carbohydrate or protein restriction, and pure C8 MCTs are particularly ketogenic (10). Similar to traditional ketogenic diets, MCT-based KDs have also been shown to reduce seizure frequencies in refractive childhood epilepsies (11). Therefore, the administration of CER-0001 may serve as a replacement for the KD, potentially offering an effective oral therapy for IS and other DEEs.

In this study, we performed a phase I, single-arm, open-label pilot trial (AC-21-024, ClinicalTrials.gov: NCT04727970) of CER-0001 for seizure control in pediatric participants with IS. Our primary objective was to evaluate the safety and tolerability of administering CER-0001 on a daily basis, with secondary objectives focused on evaluating its efficacy through clinical seizure reduction and improvements in 24 h video electroencephalogram (vEEG) recordings.

## Methods

### Eligibility

Eligible participants were infants aged between 3 and 36 months with a clinical diagnosis of IS, confirmed by a 24 h vEEG recording, who had not responded to or refused treatment with prednisolone/ACTH and vigabatrin. The participants were required to be taking no more than 3 concomitant antiseizure medications (ASMs) for at least 1 week prior to enrollment. All concomitant ASMs were required to be maintained at a stable dose for the duration of the study. Infants who were tube-fed were deemed eligible if their tube was compatible with the study medication and had a low risk of aspiration.

Participants were excluded if they had pre-existing or unstable medical conditions other than IS, abnormal laboratory and/or ECG results; known or suspected allergy to CER-0001; history of aspiration pneumonia in the past year; recent therapy with specific medications or therapies; pre-existing lethal or potentially lethal condition with a risk of death before 18 months of age; previous failure to respond to an appropriate trial of specific therapy; and known or current swallowing difficulties that posed a risk of aspiration.

### Trial design

This study was conducted in Australia and Singapore. The total study duration per participant consisted of a screening period of up to 14 days, a baseline measurement period of 7 days, 5–14 titration days, 7 maintenance days, and 7 safety follow-up days for a maximum study duration of 49 days. All eligible participants underwent a 24 h vEEG recording to establish eligibility and baseline seizure frequency on their current ASM treatment. The participants then entered a baseline measurement period where seizures/spasms were recorded in a caregiver diary by the parent/legal guardian for 7 days.

CER-0001 was titrated over a period of up to 14 days until the maximum tolerated dose (MTD) or complete seizure control was achieved for 2 consecutive days (individualized dose) or up to a maximum of 10 g/kg/day. Additional titration could be done if seizures recurred. The total daily dose of CER-0001, which provided 8 calories per gram, was divided into four doses given every 6 h. It could be taken with meals or at other times as determined by the investigator and dietician, based on an individual assessment of liquid and nutritional needs and on the total daily dose of CER-0001. Participants who showed improved seizure control after titration, in the opinion of the investigator, were maintained on the dose for at least 7 days. Participants who did not appear to be receiving clinical benefit in the opinion of the investigator underwent a repeat 24 h vEEG (by each site's blinded reader, who had undergone standardized training) on the last day of titration. If improvement was seen, they would continue in the study and receive a 7-day maintenance therapy with parental/legal guardian consent. If no improvement was seen, they could discontinue the treatment without tapering off. Safety was followed by taking notes and by a follow-up visit that took place 7 days after discontinuation. Participants who received 7-day maintenance treatment (total 12–21 days of treatment) underwent a repeat 24 h vEEG on the last day of maintenance.

Upon completion of the main phase, participants in Australia who exhibited improvement in seizure control and tolerated CER-0001 were offered continued use in a one-year open-label extension phase. In Singapore, CER-0001 was offered as part of the special access route (SARs) Program for 1 year.

### Outcomes

The primary safety outcomes were treatment emergent adverse events (TEAEs), vital signs, laboratory tests, and the Brussels Infant and Toddler Stool Scale (BITSS), where caregivers recorded and rated daily stool consistency in a diary. The BITSS is a visual tool used to assess stool consistency in infants and toddlers, specifically those not yet toilet-trained. It consists of seven color photographs of diapers containing stools, representing a range of stool types.

The secondary outcomes assessed efficacy. vEEG endpoints included the absolute and percentage change from baseline in the total number of clusters and in total cluster duration, the absolute and percentage change from baseline in all seizure durations, the proportion of participants who were spasm- and seizure-free, and the proportion of participants with 25%, 50%, and 75% reductions from baseline in the total number of clusters and total cluster duration. Caregiver diary endpoints included the absolute and percentage change from baseline in the total number of clusters and total cluster duration, the number and percentage of participants who were spasm- and seizure-free for any 48 h period, and the number and percentage of participants who were spasm- and seizure-free for the last 48 h of the treatment period. Behavioral questionnaires were distributed to the participants, which assessed the participants’ crying and sleep patterns, awake/alertness, fussiness, and overall rating on how a particular day panned out. The proportion of participants in each response category for each question at baseline and end of the treatment period was calculated.

Other variables included the presence of hypsarrhythmia at baseline and end of the treatment period based on the Burden of Amplitudes and Epileptiform Discharges (BASED) score on the vEEG for asleep and awake periods; participants with a BASED score of 4 or 5 were considered to have hypsarrhythmia. The Vineland-3 questionnaire, to assess the participant's intellectual and developmental disabilities, was distributed at baseline, end of the titration period, and end of the maintenance period. The Caregiver Global Impression of Change (Ca-GGIC) and the Clinical Global Impression of Change (C-GIC) were used at the end of titration, end of maintenance, and end of safety follow-up.

### Statistical analysis

The study was designed as a pilot study, with a primary objective of assessing the safety and tolerability of CER-0001 in children with refractory IS. Thus, no sample size calculations were performed. We planned to enroll 8–10 participants on a preliminary basis to test the efficacy of CER-0001 in IS. All data were listed and summarized using appropriate descriptive statistics. Specifically, continuous variables were summarized using the number of observations, mean, standard deviation, coefficient of variation, median, and range as appropriate. Categorical values were summarized using the number of observations and percentages as appropriate. Analyzed populations included the full analysis set (FAS), which included all participants who received at least one dose of study treatment and had at least one post-baseline efficacy measurement (used for the assessment of all efficacy analyses), and the safety analysis set (SAF), which included all participants who received at least one dose of study treatment (used for the assessment of safety and tolerability).

Baseline and post-treatment assessments were performed for each endpoint to evaluate the impact of CER-0001 compared with the participant's own baseline status. Participants who did not have any clinical benefit during titration did not continue into the maintenance phase of the study. All treated participants, including those who discontinued because of a lack of efficacy, were included in the efficacy evaluation to avoid bias. For vEEG outcomes, the primary time point for efficacy evaluation was the end of treatment evaluation, which was the end of maintenance vEEG evaluation for participants who completed the maintenance period and the end of titration vEEG evaluation for those who did not enter the maintenance period. For the caregiver diary, baseline was the last 7 days of the screening period, end of titration was the last 7 days of the titration period, and end of maintenance was the last 7 days of the maintenance period. For cumulative endpoints, such as the total number of clusters, participants with fewer than 7 days of diary data for the study period, had their available data pro-rated to a 7 day period. Changes from baseline were calculated for the end of titration and end of maintenance periods. The best change from baseline was determined as the largest decrease from baseline or the smallest increase from baseline (if there was no decrease) across the end of titration and end of maintenance phases.

## Results

**Figure 1:**
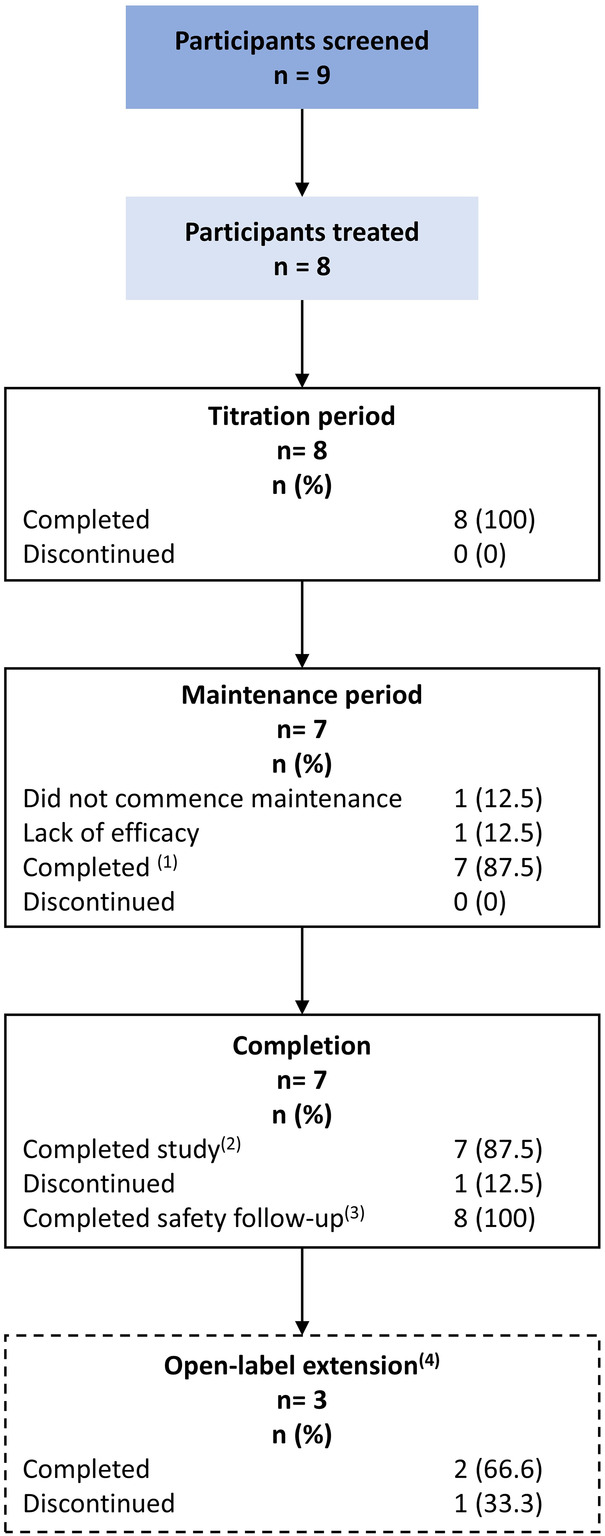
Participant disposition. (1) One participant did not take the last dose of the maintenance phase (Day 7). However, the end-of-maintenance vEEG was conducted. (2) Participants were considered to have completed the study if they completed the titration, maintenance, and safety follow-up periods. This includes one participant who discontinued treatment 1 day prior to the end of the maintenance period, but was counted as completed, since they received 6 of the 7 planned days, completed the end-of-treatment vEEG assessment, and completed the full 7-day safety follow-up period. (3) This includes one participant who completed the titration phase but did not enter the maintenance period. However, they did complete a safety follow-up following treatment discontinuation. (4) The open-label extension phase was available for Australian sites only. Two participants from Australia continued into this phase, and one participant from Singapore continued receiving CER-0001 through the Singapore Special Access Routes (SAR) Program.

This open-label pilot study was conducted between November 2021 and December 2022 at three Australian sites and one Singaporean site. Patient disposition is shown in Figure 1. A total of nine participants were enrolled, and eight entered the titration period and received study treatment for up to 2 weeks. Out of the initial eight participants, seven progressed to the maintenance phase, two continued to the open-label extension phase in Australia, and one continued receiving CER-0001 through the SAR Program in Singapore. One participant withdrew prior to the maintenance period because of a perceived lack of efficacy. All the eight dosed participants completed the safety follow-up.

The mean age of the participants was 15.1 months (range, 5–26 months), and the majority of them were male (87.5%) and of Asian descent (62.5%). The mean weight and length of the participants were 11.65 kg and 78.4 cm, respectively (Table 1). Four participants (50.0%) had cryptogenic infantile spasms and 4 had symptomatic spasms, with one having an underlying condition of trisomy 21, while 3 had underlying conditions such as genetic *TUBB2A* mutation, hemimegalencephaly and polymicrogyria, and Group B Streptococcal meningitis. According to the baseline vEEG, all treated participants had confirmed IS, while three participants also had other seizure types. The mean age at IS diagnosis was 6.25 months (range, 1–9 months), whereas the mean age at onset of first spasms was 5.50 months (range, 0–9 months). During the course of the study, all eight participants were taking at least one concomitant ASM; seven participants were taking vigabatrin, six participants topiramate, three participants levetiracetam, followed, with less frequency, by phenobarbital, valproate, and carbamazepine.

**Table 1:** Baseline demographics and infantile spasms characteristics (SAF).

Characteristic	CER-0001
	*N* = 8
Age (months)	
Mean [standard deviation (SD)]	15.1 (7.55)
Median	13.5
Min, max	5, 26
Sex, *n* (%)	
Female	1 (12.5)
Male	7 (87.5)
Race, *n* (%)	
Caucasian	2 (25)
Asian	5 (62.5)
Indian/Indian Subcontinent	1 (12.5)
Chinese	2 (25.0)
Vietnamese	1 (12.5)
Other Southeast Asian	1 (12.5)
Aboriginal Australian	1 (12.5)
Weight (kg)	
Mean (SD)	11.65 (2.768)
Median	10.97
Min, max	8.53, 16.70
Length (cm)	
Mean (SD)	78.4 (8.98)
Median	75.2
Min, max	68.0, 90.0
Type of Infantile spasms, *n* (%)	
Cryptogenic	4 (50.0)
Symptomatic	4 (50.0)
Underlying conditions, *n* (%)	
Tuberous sclerosis complex (TSC)	0 (0)
Trisomy 21	1 (12.5)
Focal structural abnormality	0 (0)
Perinatal hypoxic-ischemic encephalopathy	0 (0)
Other	3 (37.5)
Genetic Tubb2a mutation	1 (12.5)
Hemimegalencephaly and Polymicrogyria	1 (12.5)
Group B Streptococci meningitis	1 (12.5)
Seizure type,^a^ *n* (%)	
Epileptic spasms^b^	7 (87.5)
Focal onset—aware—motor onset	1 (12.5)
Focal onset—impaired awareness—motor onset	3 (37.5)
Unclassified—tonic—stiffening and clenching of fists, gritting of teeth	1 (12.5)
Unknown onset—motor—other motor	1 (12.5)
Age at diagnosis (months)	
Mean (SD)	6.25 (2.6)
Median	6.50
Min, max	1.0, 9.0
Age at onset of first symptoms (months)	
Mean (SD)	5.50 (4.4)
Median	6.5
Min, max	0.0, 9.0

^a^ Participants can be included more than once.

^b^ Data reported in line with the IS history reported by site on the eCRF. Baseline vEEG data confirmed that all eight treated participants had epileptic spasms at baseline.

### Exposure

CER-0001 was administered to all eight participants during the titration phase, with seven participants continuing into the maintenance period. One participant stopped taking CER-0001 during the maintenance period because of a lack of perceived efficacy. The average time of exposure during titration was 12.09 days (range, 6.5–14.75 days). During maintenance, the average exposure time was 6.93 days (range, 5.5–7.5 days). The highest dose reached varied between 3.41 and 6.47 g/kg/day, with two participants reaching 60% of their daily caloric intake provided by CER-0001 (Table 2). Of the seven participants who progressed to the maintenance phase, five underwent dose escalation that exceeded the intended titration window.

**Table 2:** CER-0001 dosing and *β*-hydroxybutyrate levels.

Participant	Age at diagnosis (months)	Age at screening (months)	Baseline weight (kg)	Number of days of titration	Days on IMP (titration + maintenance)	IMP exposure (days)	Final dose at the end of the maintenance period	Highest dose reached	Highest blood βHB level^a^
001	9	12	9.6	14	21	20	9 mL QID = 40% 3.78 g/kg/day	12 mL QID = 55% 5 g/kg/day	1.03 mmol/L Dose 2 1h09 m after IMP
002	1	5	9.5	14	20	19.25	9 mL QID = 34% 3.6 g/kg/day	11 mL QID = 40% 4.53 g/kg/day	2.97 mmol/L Dose 1 1h30 m after IMP
003	5	13	9.5	14	15 (did not enter the maintenance phase)	14.75	13 mL QID = 43% (titration period) 5.39 g/kg/day	15 mL QID = 50% 6.47 g/kg/day	1.38 mmol/L Dose 1 1h54 m after IMP
004	7	23	11.9	7	15	14	11 mL QID = 40% 3.52 g/kg/day	11 mL QID = 40% 3.64 g/kg/day	0.9 mmol/L Dose 2 5 h after IMP
005^b^	9	14	12.9	14	22	21	8 mL QID = 60% 2.42 g/kg/day	10.5 mL QID = 60% 3.41 g/kg/day	1.3 mmol/L Dose 2 3h58 m after IMP
006	6	21	16.7	15	23	22.25	20 mL QID = 60% 4.88 g/kg/day	20 mL QID = 60% 4.79 g/kg/day	3.1 mmol/L Dose 1 3h11 m after IMP
007	6	26	13.2	14	21	20.5	13 mL QID = 40% 3.74 g/kg/day	13 mL QID = 40% 3.74 g/kg/day	1.91 mmol/L Dose 4 17h08 m after IMP
008	7	7	8.5	7	14	13.5	11 mL QID = 50% 5.13 g/kg/day	11 mL QID = 50% 5.18 g/kg/day	2.8 mmol/L time unknown after IMP

^a^ Sample collected randomly at the end of the titration period or at the end of the maintenance period as part of the safety laboratory.

^b^ This participant was on a reduced calorie diet (due to weight gain from prior steroid treatment), and as dosing is done based on the percentage of daily calorie intake, calorie intake was increased during the study; therefore, the g/kg/day amount increased (the original 60% dose ranged from 8 mL QID to 10 mL QID). As an estimate, a participant of that weight may have a 40%–50% higher calorie requirement.

The participants were adherent to dosing. Table 3 presents the dosing log data. Seven participants continued into the maintenance phase, and of these, five continued to escalate beyond the end of their titration period, including 4 participants who titrated over the protocol-defined maximum period of 14 days. Some participants continued to titrate during the maintenance phase. The rate of adherence to the dosing was generally above 90% for the participants.

**Table 3:** Dosing.

Participant	Titration phase		Maintenance phase	
	Planned doses	Administered	Planned doses	Administered
		*N* (%)		*N* (%)
001	56	54 (96.4)	28	26 (92.9)
002	56	55 (98.2)	28	22 (78.6)
003	61	61 (100.0)	NA^a^	NA^a^
004	28	26 (92.9)	30	30 (100.0)
005	56	54 (96.4)	30	30 (100.0)
006	60	59 (98.3)	30	30 (100.0)
007	56	54 (96.4)	28	28 (100.0)
008	28	26 (92.9)	28	28 (100.0)

^a^ Participant did not enter the maintenance phase.

### Safety and tolerability

All participants experienced at least one treatment-emergent adverse event (TEAE), five participants (62.5%) reported at least one TEAE that was considered related to CER-0001 by the investigator, and five experienced at least one moderate to severe TEAE. The most common system organ class was gastrointestinal (GI) disorder, with 6 (75%) participants experiencing at least one GI TEAE and four participants (50%) experiencing at least one GI TEAE that was considered causally related to CER-0001. Vomiting was the most frequent TEAE in the study, experienced by four participants (two mild and two moderate in severity) and all considered related to IMP. Furthermore, one participant required a nasogastric tube because of an oral aversion TEAE. Weight/BMI was monitored, with no significant changes recorded. Two participants experienced mild neutropenia, one of which was considered causally related to CER-0001. An exploratory micronutrient panel (Mg, Zn, P, Selenium, and Cu) showed that two participants had high predose serum copper levels and five participants had high copper levels at least once after baseline, although four out of these five had an elevation 8 days after stopping treatment and 1 one day after stopping treatment. Formula, food, and water tests were not conducted for copper; however, a testing of CER-0001 revealed that it had low levels of copper.

Three participants experienced serious adverse events (SAEs) during the study. Two reported SAEs during the maintenance phase (aspiration, considered related to CER-0001; and rhinovirus bronchiolitis, considered not related to CER-0001) and one experienced two SAEs during the safety follow-up (COVID-19 and irritability, both considered not related to CER-0001). Only one participant had to discontinue treatment because of a SAE of rhinovirus bronchiolitis during the maintenance phase. Although the participant missed 1 day of dosing in the maintenance period, it was still considered that the participant had completed the period because only one missed day denoted almost 100% completion; the completion was justified also because the participant had the end-of-treatment vEEG performed. The long-term safety data on three participants up to 1 year were reported as satisfactory.

The study found no consistent changes in hematology and clinical chemistry measurements, vital signs, or ECGs. No significant changes were observed in BITSS, participant behavior questionnaire scores, or Vineland-3 questionnaire scores.

### Efficacy

For vEEG data, the mean daily total number of clusters decreased from 8.4 to 6.9 between baseline and the end of treatment. This resulted in a mean reduction of 16.6% and a median percentage change of −25%. In addition, the mean total cluster duration decreased from 6,877.3 to 5,651.9 s (circa 115–94 min), with a mean percentage change of 44.4% from baseline and a median percentage change of −23.1% from baseline (Table 4). Four participants (50%) experienced a reduction of at least 50% from baseline in the total number of clusters at the end of treatment, including two participants (25%) with a 100% reduction and one participant with a reduction ≥75% from baseline (Table 5). The total duration of clusters and the total seizure duration endpoints were highly variable, leading to large differences between participants and large discrepancies between the mean and the median change from baseline. The mean and percentage changes from baseline for both endpoints were heavily influenced by a single value, resulting in a mean increase from baseline and a median decrease from baseline.

**Table 4:** Summary of absolute values and change and percentage change from baseline in the total number of clusters and total cluster duration—vEEG (FAS).

Parameter	Statistic	Baseline	End of treatment	Change from baseline	% Change from baseline
Total number of clusters	n Mean (SD) Median Min, max	8 8.4 (6.91) 5.0 2, 19	8 6.9 (9.88) 2.5 0, 27	8 −1.5 (6.21) −0.5 −13, 8	8 −16.6 (84.68) −25.0 −100, 150
Total cluster duration	n Mean (SD) Median Min, max	8 6,877.3 (14,261.77) 604.0 48, 41,580	8 5,651.9 (14,489.70) 182.5 0, 41,460	8 −1,225.4 (3,029.09) −71.0 −7143, 1,840	8 44.4 (201.09) −23.1 −100, 511

**Table 5:** Derived 24 h vEEG cluster parameters (FAS).

Participant	Number of clusters		Total cluster duration (s)		Total number of seizures		Total seizures duration	
	Baseline, end of treatment	Change from baseline (% change)	Baseline, end of treatment	Change from baseline (% change)	Baseline, end of treatment	Change from baseline (% change)	Baseline, end of treatment	Change from baseline (% change)
001	17, 17	0 (0.0)	41,580, 41,460	−120 (−0.3)	0, 0	0 (0.0)	0, 0	0 (0.0)
002	13, 0	−13 (−100.0)	4,620, 0	−4,620 (−100.0)	0, 0	0 (0.0)	0, 0	0 (0.0)
003	19, 27	8 (42.1)	57, 81	24 (42.1)	0, 0	0 (0.0)	0, 0	0 (0.0)
004	2, 1	−1 (−50.0)	636, 284	−352 (−55.3)	11, 0	−11 (−100.0)	1,832, 0	−1,832 (−100.0)
005	2, 5	3 (150.0)	572, 1,162	590 (103.1)	0, 0	0 (0.0)	0, 0	0 (0.0)
006	6, 0	−6 (−100.0)	7,145, 2	−7,143 (−100.0)	3, 0	−3 (−100.0)	15, 0	−15 (−100.0)
007	4, 1	−3 (−75.0)	360, 2,200	1,840 (511.1)	0, 0	0 (0.0)	0, 0	0 (0.0)
008	4, 4	0 (0.0)	48, 26	−22 (−45.8)	227, 107	−120 (−52.9)	565, 252	−313 (−55.4)

Two participants (25%) were spasm- and seizure-free based on the end of treatment vEEG. No participant developed new seizure types during the trial. Of the eight participants in the FAS, three experienced other seizure types at baseline based on the 24 h baseline vEEG. Two of the three participants with other seizure types at baseline had complete resolution of their other seizure types, and the third had a 55% reduction from baseline in the other seizure types.

**Figure 2:**
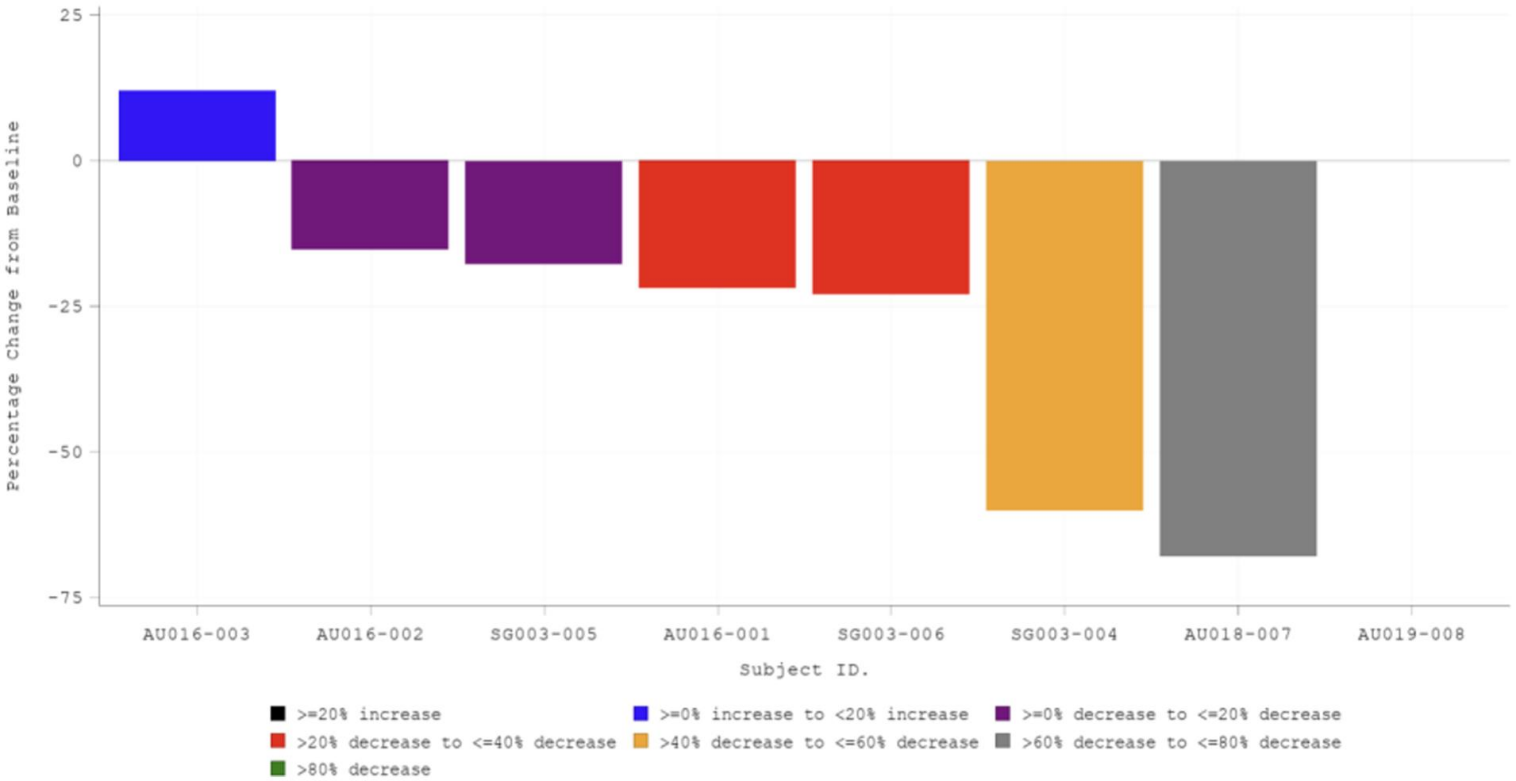
A waterfall plot of maximum percentage change from baseline in the total number of clusters reported in caregiver diaries. Best percentage change from baseline based on caregiver diaries. Participant numbers are shown on the *X*-axis, indicating site location as AU for Australia or SG for Singapore followed by participant number. Best percentage change is defined as the biggest decrease (at the end of the titration period or at the end of the maintenance period). If there were no decreases at either period, the smallest increase was used. If a participant did not enter the maintenance phase, the value at the end of titration phase was used. No clusters were reported during the baseline period for participant AU019-008.

At baseline, seven out of eight participants had clusters as reported in the caregiver diary, with a mean of 49.0 clusters. After titration, the number decreased to 41.8, a change of −7.2 and a favorable percentage change of −23.4% from baseline, while at the end of the maintenance phase, the mean number of clusters was 49.6, a change of 0.6 and a change of −7.1% from baseline: a waterfall plot of the best percentage change from baseline as reported in caregiver diaries revealed that six of the seven participants with epileptic spasms at baseline experienced a reduction from baseline in the number of clusters, including two participants with a reduction of greater than 50% from baseline (Figure 2).

The baseline mean total cluster duration was 168.2 s; after titration, it was 224.1 s, a change of 55.9 s, a mean percentage change of 15.5% from baseline, and a median percentage change of −13.0 from baseline; at the end of the maintenance phase, the mean total cluster duration was 428.4 s, a change of 260.2, a percentage change of 88.6% from baseline, and a median percentage change of 16.5 from baseline. Three participants (37.5%) remained spasm- and seizure-free for at least one 48 h period, while one participant (12.5%) remained seizure-free during the last 48 h treatment period. The difference between the median mean and median is due to a single very large outlier, and with the exception of this one participant, most participants did not experience much change in the total cluster duration, according to the caregiver diary.

No consistent trends in the local site readings of BASED scores, whether participants were awake or sleeping, were identified. While sleeping, three participants showed improvement from baseline, the condition worsened in two participants, and in the other three, the condition remained unchanged. In the awake state, the condition of two participants worsened, it improved in four participants, and in two, it remained unchanged.

The majority of caregivers (6/8) reported improvement in Ca-GIC scores in participants during the titration period, and in all seven participants who continued into the maintenance period, the caregivers reported improvements at the end of the maintenance treatment. Similarly, at the end of the titration period, five out of eight clinicians reported improvement in C-GIC scores in participants, with six out of seven clinicians reporting that the participants improved during the maintenance period. Based on both the caregiver (Ca-GIC) and the clinical (C-GIC) ratings, one participant was reported to be minimally worse during the titration period. This was the same participant in whom treatment was discontinued at the end of the titration period and who did not enter the maintenance phase. Two participants who continued up to a year were reported to have satisfactory efficacy, although no standard scales were used.

## Discussion

This study provided evidence supporting the feasibility, safety, and potential efficacy in using CER-0001 for treating IS. There were no dropouts in the titration period, and seven out of the eight infants were judged by the investigator to have benefited enough to continue into the maintenance treatment phase. This suggests that there was an overall acceptability of the treatment among the study population. Moreover, 2 participants continued to receive CER-0001 up to a year, with safety and efficacy reported as satisfactory.

An analysis of dosing diaries showed that the 14-day titration period for CER-0001 dosing may have been too short for infants to reach their MTD. A total of 71% of participants increased their doses beyond the titration period, but only two out of the eight reached the maximum dose allowed (60% of daily caloric intake). Doses by the end of the maintenance period ranged from 34% to 60% of caloric intake. Interestingly, one participant saw reduced cluster episodes at 34% caloric intake, suggesting that 60% caloric intake may not necessarily indicate effectiveness.

CER-0001 was found to be generally safe and well-tolerated, with GI TEAEs being the most commonly reported in six participants (75%). The only other TEAEs reported in more than one participant, considered to be caused by CER-0001 treatment, were mild copper elevation in two participants. A comprehensive safety data review was conducted, which included an external literature review and analysis of all medications and dietary supplements taken, to determine the reason for the increase in copper levels. The findings revealed that the increase could be attributed to several factors, including the administration of multivitamins, as well as other external sources such as water, food, and formula. In addition, inherent variation in copper levels and assay norms, particularly in very young individuals and those with epilepsy, could also contribute. While the exact cause remains unknown, it was determined that the study drug administration is unlikely to be linked to the elevated copper levels given that the levels of copper in CER-0001 are low and there is no correlation between the dose or time of administration and the increase in copper levels. Future studies will monitor these copper levels. No other findings have been discovered that could potentially compromise the safety of the participants. The safety profile of CER-0001 continues to be favorable when compared with the side effects of first-line treatments, such as hypertension, irritability, infection, and cerebral atrophy for hormonal therapies and retinal toxicity for vigabatrin (12).

The ultimate goal of treatment is complete cessation of spasms and resolution of hypsarrhythmia on EEG, as these are associated with the best developmental outcomes. However, partial responses such as a 50% reduction are still considered clinically relevant, particularly when complete remission is not immediately achievable (13). In the present study, 50% of the participants experienced a reduction of over 50% in the total number of clusters on the 24 h vEEG: two showed a 100% reduction from baseline, while one experienced a decrease of over 75%. In addition, a minimum of 50% reduction in the total cluster duration was observed in 37.5% of the participants, with one participant seeing a 100% reduction and another experiencing a reduction of more than 99.9%. Among the three participants who did exhibit other seizure types at baseline, there was a reduction of at least 50% in their other seizure types compared with baseline. Among them, two participants showed a 100% reduction from baseline. The observed positive effects on additional seizure types indicate the potential benefits of CER-0001 beyond epileptic spasms. This aspect should be considered for evaluation in any forthcoming clinical trials.

The caregiver diary data regarding cluster counts, cluster durations, and all-seizure activity did not exhibit any discernible patterns. A comparison of the caregiver diary and vEEG data from the same 24 h period revealed a lack of concordance. There are inconsistencies present within the data recorded in the caregiver diary, making it challenging to draw definite conclusions from it, although the number of clusters seems to have slightly improved and the mean duration of spasms appears to have worsened. It is also important to note that the diary can be burdensome for caregivers; hence, the amount of diary data should be evaluated carefully, and the endpoints should be limited to those that can easily and consistently be assessed. The high variability and inconsistencies in caregiver-reported spasm diaries likely stemmed from challenges in accurately identifying infantile spasms, which are often subtle and easily confused with normal movements. Additional factors include caregiver fatigue, inconsistent diary use, recall bias, and poor instructions. Variability may also reflect true fluctuations in seizure frequency or differences in interpretation across caregivers.

There are some important considerations to keep in mind when enrolling participants in future trials. One useful tool that could be utilized is diary data to eliminate participants who do not experience regular spasms during screening, although study enrollment should depend on spasms that are confirmed by vEEG. It is important to note that including participants with very low cluster counts at baseline may introduce noise in both directions, making it challenging to detect true differences. To alleviate this issue and enhance the trial's sensitivity in quantifying the true treatment effect, it might also be helpful to introduce a minimum threshold for the number of baseline clusters and entry duration. As shown in Table 2, peak β-hydroxybutyrate (BHB) levels between approximately 1.0 and 3.1 mmol/L indicate that participants consistently achieved nutritional ketosis, although at levels lower than those typically seen with classical ketogenic diets, and exploratory analyses suggested a relationship between higher BHB levels and seizure reduction despite irregular sampling time points.

Previous clinical trials and treatment guidelines around current second-line IS therapies do not provide strong evidence for the use of a variety of traditional ASMs, including levetiracetam, topiramate, zonisamide, valproic acid, felbamate, and several benzodiazepines such as nitrazepam (14–20). A comparative study of KD versus standard ASMs showed that less than 10% of participants had a complete response and less than 20% experienced a reduction of 59% in spasms. In contrast, with CER-0001 treatment, 4 participants (50%) experienced a reduction from baseline of at least 50% in the total number of clusters at the end of treatment, including 2 participants (25%) with a 100% reduction. Such outcomes can be viewed as a first step toward the development of a potential novel ketogenic drug for use as a secondary or even first-line treatment option (9). This is especially crucial in DEEs such as IESS, where early and safe treatment may play a role in improving outcomes (4, 21, 22).

## Conclusion

CER-0001 was administered to eight infants, all of whom completed the initial study. By the end of the titration period, seven infants had been considered to have received clinical benefit and they continued to receive maintenance treatment, with three continuing up to a year. The study limitations include the small sample size, majority male population, and high variability in the efficacy results. Nonetheless, the results show that the treatment has an accepted tolerability profile, and a promising signal of efficacy was detected, with 50% of participants (4/8) achieving at least a 50% reduction in spasm cluster frequency, of whom two (25%) showed complete resolution based on the end-of-treatment vEEG. The National Infantile Spasms Consortium (NISC) and global neurology associations have found poor responses to infantile epileptic spasm syndrome (IESS) treatments beyond first-line standard agents and KD trials (14). The encouraging results from this trial for a medication not requiring a KD warrant further evaluation in a larger controlled study. To confirm these findings and better define the clinical benefit across diverse patient populations, a multicenter, randomized Phase 2/3 trial is required.

## Data Availability

The raw data supporting the conclusions of this article will be made available by the authors upon reasonable request, without undue reservation.
